# The Predictive Role of Preoperative Malnutrition Assessment in Postoperative Outcomes of Patients Undergoing Surgery Due to Gastrointestinal Cancer: A Cross-Sectional Observational Study

**DOI:** 10.3390/jcm13237479

**Published:** 2024-12-09

**Authors:** Eva Karanikki, Maximos Frountzas, Irene Lidoriki, Alexandros Kozadinos, Adam Mylonakis, Iliana Tsikrikou, Maria Kyriakidou, Orsalia Toutouza, Efthimios Koniaris, George E. Theodoropoulos, Dimitrios Theodorou, Dimitrios Schizas, Konstantinos G. Toutouzas

**Affiliations:** 1 Department of Clinical Nutrition, Hippocration General Hospital, 11527 Athens, Greece; e.karanikki@hippocratio.gr; 2 1st Propaedeutic Department of Surgery, Hippocration General Hospital, National and Kapodistrian University of Athens, 11527 Athens, Greece; georgetheocrs@live.com (G.E.T.); dimitheod@netscape.net (D.T.); tousur@hotmail.com (K.G.T.); 3 Department of Environmental, Occupational Medicine and Epidemiology, Harvard School of Public Health, Boston, MA 02115, USA; elidoriki@hsph.harvard.edu; 4 1st Department of Surgery, Laikon General Hospital, National and Kapodistrian University of Athens, 15784 Athens, Greece; akozadinos@med.uoa.gr (A.K.); adam.mylonakis@gmail.com (A.M.); il.tsikrikou@gmail.com (I.T.); maria.ath.2007@gmail.com (M.K.); schizasad@gmail.com (D.S.); 5 Medical School, Imperial College, London SW7 2AZ, UK; orsalia.toutouza20@imperial.ac.uk; 6 Department of Pathology, Hippocration General Hospital, 11527 Athens, Greece; e.koniaris@hippocratio.gr

**Keywords:** malnutrition, surgery, postoperative, PG-SGA, GLIM, GNRI, CONUT, colorectal cancer, hepato-pancreato-biliary cancer, upper gastrointestinal cancer, surgery, postoperative, outcomes

## Abstract

**Background:** Malnutrition affects patients undergoing surgery for gastrointestinal cancers and contributes to poor postoperative outcomes, including increased complication rates, longer hospital stays, and higher mortality. Despite the availability of several malnutrition screening tools and prognostic scores, their effectiveness in predicting postoperative outcomes remains unclear. This study aimed to compare the predictive accuracy of Patient-Generated Subjective Global Assessment (PG-SGA), Global Leadership Initiative on Malnutrition (GLIM), Geriatric Nutritional Risk Index (GNRI), and Controlling Nutritional Status (CONUT) score for postoperative outcomes in patients undergoing surgery for colorectal, hepato-pancreato-biliary and upper gastrointestinal cancers. **Methods:** A cross-sectional observational study from March 2022 to October 2023 was conducted in two university surgical departments, after registration on ClinicalTrials database (NCT05795374). Patient characteristics, preoperative nutritional status and postoperative outcomes were analyzed. **Results:** In total, 480 patients were enrolled. CONUT and GNRI demonstrated high specificity (over 90% and 80%, respectively) for predicting overall complications, major complications, prolonged hospital stay, mortality, and advanced disease stage across all cancer types. Notably, CONUT showed a specificity over 97% and GNRI over 89.7% for colorectal and upper gastrointestinal cancer patients, respectively, despite their lower sensitivity. On the contrary, PG-SGA and GLIM presented better sensitivity (up to 50%), but slightly lower specificity (up to 86.4%). **Conclusions:** CONUT and GNRI are valuable for ruling out non-at-risk patients for adverse postoperative outcomes, while PG-SGA and GLIM provide better sensitivity. A step-up approach—initial screening with PG-SGA and GLIM, followed by detailed evaluation with CONUT or GNRI— should be validated in future studies across diverse clinical settings.

## 1. Introduction

According to the World Health Organization (WHO), malnutrition is a significant contributor to global mortality, particularly among children under 5 years of age, leading to nearly half of all deaths in this age group. In 2022, an estimated 149 million children under five were stunted (low height for age) and 45 million were wasted (low weight for height) [1]. Malnutrition still remains a critical concern in patients undergoing surgery for gastrointestinal cancers, including colorectal (CRC), hepato-pancreato-biliary (HPB) and upper gastrointestinal (GI) cancers [2]. It can be detected in at least 50% of patients with gastrointestinal cancer who undergo surgery, and it significantly impacts postoperative outcomes and complication rates, as well as patients’ recovery, functional status and overall survival [3,4,5,6,7]. Malnutrition in such patients is often underdiagnosed and if left undertreated, it could lead to adverse postoperative results, such as increased infection rates, delayed wound healing, prolonged hospital stay, and higher postoperative mortality [6,8,9,10,11,12]. Furthermore, malnutrition diagnosis, high malnutrition risk scores according to screening tools, as well as sarcopenia, were identified as prognostic factors for postoperative complications and/or survival in gastrointestinal cancer patients [13,14,15,16]. Under these circumstances, the importance of accurate malnutrition screening and assessment in such patients becomes very important.

Despite significant research efforts, the effectiveness of several malnutrition screening tools in predicting postoperative outcomes in gastrointestinal cancer patients is still unclear. So far, large variability is observed in the literature regarding sensitivity and specificity of several screening tools in detecting malnutrition [17,18], but only a few studies were conducted for investigating the diagnostic accuracy metrics of malnutrition screening tools regarding postoperative results [19,20,21,22,23]. Some of the malnutrition assessment tools that were investigated include the Patient-Generated Subjective Global Assessment (PG-SGA), the Global Leadership Initiative on Malnutrition (GLIM) criteria, the Geriatric Nutritional Risk Index (GNRI) and the Controlling Nutritional Status (CONUT) score. Whereas each tool has been validated in different clinical settings, their comparative performance in predicting postoperative results among gastrointestinal cancer patients remains inadequately explored [24,25].

The aim of this study is to evaluate the predictive accuracy of these malnutrition assessment tools for the postoperative outcomes of patients operated due to gastrointestinal cancers. Specifically, we seek to determine the sensitivity, specificity, and the positive and negative predictive value of PG-SGA, GLIM, GNRI, and CONUT in estimating overall complications, major complications, length of hospital stay, mortality, and advanced disease stage in this patient population. By understanding the prognostic power of each tool in the different GI cancer types, clinicians could make informed decisions on nutritional management, ultimately improving patient care and postoperative results. This research could also contribute to the ongoing efforts of refining malnutrition screening processes and enhancing the quality of life for patients with gastrointestinal cancers.

## 2. Materials and Methods

### 2.1. Ethical Approvals

We conducted a cross-sectional, observational study at two university surgical departments, from March 2022 until October 2023. The scientific protocol was approved by the Institutional Review Board of the Hippocration General Hospital of Athens (Ref. No. 3975/4-3-2022) and was registered at the international database ClinicalTrials.gov (Id. No. NCT05795374). It was conducted in compliance with the Declaration of Helsinki guidelines about ethical principles for medical research involving human subjects. A written informed consent was obtained by all patients before participation in the study.

### 2.2. Patient Population

Four hundred and eighty patients undergoing surgery for colorectal (CRC), hepato-pancreato-billiary (HPB) and upper GI cancer, aged 18 years or older were prospectively enrolled. People who declined to take part in this study were excluded from participation. Included patients were monitored during their hospital stay and at least 30 days after their discharge.

### 2.3. Data Collection

Patient characteristics including gender, age, weight, height, body mass index (BMI), American Society of Anesthesiologists (ASA) physical status classification, and Eastern Cooperative Oncology Group (ECOG) Performance Status scale were reported. Postoperative outcomes, such as the presence of overall complications, major complications (Clavien-Dindo ≥ 3), 30-day mortality, length of stay (LOS), and advanced disease stage (≥3) were collected.

### 2.4. Nutritional Assessment

#### 2.4.1. PG-SGA

In the current study, we used the culturally adapted and validated Greek version of the PG-SGA [26]. The PG-SGA (Patient-Generated Subjective Global Assessment) classifies patients into three categories: (A) well-nourished; (B) moderately malnourished/at risk of malnutrition; or (C) severely malnourished. Currently, the PG-SGA is the preeminent interdisciplinary patient assessment (weight, intake, symptoms, functional status, disease state, metabolic stress, and nutritional physical examination) in oncology and other chronic catabolic conditions. The scored PG-SGA includes the four patient-generated historical components (weight history, food intake, symptoms and activities and function—also known as the PG-SGA Short Form), the professional part (diagnosis, age, metabolic stress, and physical exam), the Global Assessment (A = well nourished, B = moderately malnourished or suspected malnutrition, C = severely malnourished), the total numerical score, and the nutritional triage recommendations. Subsequently, the scored PG-SGA allows for triaging of specific nutrition interventions, as well as facilitating quantitative outcome data collection. For data analysis purposes, patients were divided into two categories, patients with PG-SGA A and B and patients with PG-SGA C.

#### 2.4.2. GLIM Criteria

GLIM (Global Leadership Initiative on Malnutrition) offers a framework for diagnosing malnutrition in adult patients. It includes five practicable indications that can be used in routine clinical practice. To diagnose malnutrition using the GLIM criteria, weight changes within six months (%) and body mass index (BMI) were calculated using patients’ weight history and height (Supplementary Table S1). Based on the GLIM criteria, participants who had a combination of at least one phenotypic criterion (weight loss and/or low BMI for age and/or reduced muscle mass mainly using calf circumferences) and one etiologic criterion (disease burden and inflammatory condition of cancer) were categorized as malnourished. An assessment of muscle function using hand grip strength was used as a supportive measure in the GLIM consensus. Malnourished patients were categorized based on the severity of malnutrition into Stage 1 (moderate) and Stage 2 (severe) (Supplementary Table S2). The remaining participants were categorized as well-nourished [27,28].

#### 2.4.3. GNRI

The Geriatric Nutritional Risk Index (GNRI), based on serum albumin levels, present body weight, and ideal body weight, is a simple screening tool to assess nutritional risk. Baseline GNRI was calculated from serum albumin and BMI was obtained at the first hospital visit, using the following formula [29]: GNRI = 14.89 × serum albumin (g/dL) + 41.7 × (present body weight/ideal body weight). If the patient’s body weight exceeded the ideal body weight, the present body weight/ideal body weight was set to 1. The ideal body weight was defined as the value calculated from height and a BMI of 22 kg/m^2^, instead of calculations using the Lorentz formula in the original GNRI equation [30,31]. Patients were divided into four groups according to the GNRI: G0 (no risk, >98), G1 (low risk, 92–98), G2 (moderate risk, 82–91), and G3 (high risk, <82) [29,32]. For data analysis purposes, patients were divided into two categories based on their GNRI scores: those with a GNRI ≥ 82, indicating no to moderate risk and those with a GNRI < 82, indicating major risk.

#### 2.4.4. CONUT

The Controlling Nutritional Status (CONUT) score was calculated using serum albumin concentration, peripheral lymphocyte count and total cholesterol concentrations (Supplementary Table S3). In brief, each parameter was scored as follows: albumin concentration: ≥3.5 mg/dL: 0 points, 3.0–3.49 mg/dL: 2 points, 2.5–2.99 mg/dL: 4 points, and <2.5 mg/dL: 6 points. Total lymphocyte count: ≥1600/mm^3^: 0 points, 1200–1599/mm^3^: 1 point, 800–1199/mm^3^: 2 points, and <800/mm^3^: 3 points. Total cholesterol levels were scored as: ≥180 mg/dL: 0 point, 140–179/mm^3^: 1 point, 100–139/mm^3^: 2 points, and <100/mm^3^: 3 points. The sum of these scores was defined as the CONUT score [33,34].

### 2.5. Statistical Analysis

A statistical analysis was conducted using the SPSS version 22.0 (SPSS Inc., Chicago, IL, USA). The Kolmogorov–Smirnov test was used to check the normality of distributions among quantitative variables. Mean values and standard deviations (SD) were used for normally distributed outcomes, while medians and interquartile ranges were used for abnormally distributed outcomes. Absolute (N) and relative (%) frequencies were used to describe qualitative variables. The validity of different screening tools was evaluated by weighing sensitivity, specificity, positive predictive value (PPV), and negative predictive value (NPV) for the reported postoperative outcomes. In addition, the receiver operator characteristic (ROC) curve was applied to establish the predictive power of each malnutrition screening tool. The area under the curve (AUC) with no discrimination power is 0.5, while values over 0.8 indicate excellent discrimination and a value from 0.6 to 0.8 meaning clinically useful parameters. A post hoc power analysis was conducted considering significance level (alpha) at 0.05, which indicated a post hoc power of 85% [35].

## 3. Results

### 3.1. Baseline Characteristics

In total, 480 patients undergoing surgery due to gastrointestinal cancer were included in this bi-center observational study. Colorectal cancer was present in 205 patients with a mean age of 69.2 ± 11.7 years old, 103 patients suffered from HPB cancer with a mean age of 63.8 ± 12.4 years old, and 172 patients had upper GI cancer with a mean age of 66.6 ± 11.2 years old. Among patients with colorectal cancer, 62% were male, while the proportion of male patients was 67% for both HPB cancer and upper GI cancer (Table 1). Additionally, 33.67% of patients undergoing surgery for colorectal cancer had a normal BMI, compared to 35.71% of HPB cancer patients and 39.29% of upper GI cancer patients. In terms of ECOG status, nearly 60% of colorectal cancer patients had a score of 0, whereas 51% of HPB cancer patients and 55.8% of upper GI cancer patients also demonstrated an ECOG status of 0. Finally, an ASA score of 1 was observed in 29.9% of patients undergoing surgery for colorectal cancer, 24.5% for HPB cancer, and 28% for upper GI cancer.

### 3.2. Colorectal Cancer

The malnutrition assessment according to the PG-SGA tool indicated that 21.5% of colorectal cancer patients were severely malnourished (Stage C, Table 2). Furthermore, the GLIM tool demonstrated a similar rate of severe risk for malnutrition (19.1%). However, GNRI and CONUT nutritional screening scores identified lower rates of severe nutritional risk (4.9% and 1.06%, respectively, Table 1).

The CONUT score presented specificity above 97.5% for predicting various outcomes, including 100% for overall complications, 97.5% for major complications (Clavien-Dindo ≥ 3), 100% for greater LOS, 98.9% for higher mortality, and 100% for advanced disease stage (≥3). In addition, the specificity of GNRI was above 95.5% in predicting the aforementioned postoperative outcomes. In detail, the specificity for GNRI in predicting overall complications was 96.2%, major (Clavien-Dindo ≥ 3) complications was 95.5%, greater LOS was 97.7%, higher mortality was 96%, and advanced disease stage (≥3) was 97.8%. The PG-SGA and GLIM scores had lower specificity, ranging from 78% to 86.4% and 78% to 81.8%, respectively, for predicting postoperative outcomes. The CONUT score had 100% PPV for predicting complications, greater LOS, and advanced disease stage, whereas GNRI had 90% PPV for predicting greater LOS. Furthermore, both PG-SGA and GNRI presented a sensitivity of 50% in determining patient mortality. GLIM demonstrated a strong predictive ability for mortality among colorectal cancer patients (AUC 0.601) compared to PG-SGA (AUC 0.356), GNRI (AUC 0.258), and CONUT (AUC 0.505).

### 3.3. HPB Cancer

The malnutrition screening assessment using the PG-SGA tool indicated that 24.24% of HPB cancer patients were at severe risk of malnutrition (Stage C, Table 3). Furthermore, the GLIM tool demonstrated a similar rate of severe risk for malnutrition (22.68%). However, the GNRI demonstrated that a large portion of patients (89.22%) were at major nutritional risk, while the CONUT score identified a very low rate of severe nutritional risk (3.29%, Table 2).

The CONUT score demonstrated the highest specificity, ranging from 90% to 100% in predicting several postoperative outcomes (Table 3). In detail, the CONUT score showed 100% specificity for overall complications, 96.8% for major complications (Clavien-Dindo ≥ 3), 90% for greater length of stay (LOS), 98.8% for higher mortality, and 100% for advanced disease stage (≥3). GNRI’s specificity ranged from 90% to 93.5% for these outcomes, with specificities of 93.5% for overall complications, 90% for higher LOS and 90.2% for higher mortality. However, GNRI’s specificity for predicting major complications and advanced disease stage was lower than the CONUT score (80%).

The PG-SGA and GLIM scores had lower specificity, with ranges of 50% to 85.7% and 60% to 80.7%, respectively, for predicting postoperative outcomes. In terms of positive predictive value (PPV), the CONUT score had a PPV of 100% for complications and advanced disease stage, while GNRI had a PPV of 90.9% for greater LOS. Sensitivity was 55.6% for GLIM and 50% for PG-SGA in predicting higher mortality. Regarding the area under the curve (AUC), PG-SGA exhibited a strong predictive ability for LOS among HPB cancer patients (AUC 0.632) compared to GLIM (AUC 0.588), GNRI (AUC 0.517), and CONUT (AUC 0.543).

### 3.4. Upper GI Cancer

The malnutrition screening assessment according to the PG-SGA tool indicated that 29.76% of colorectal cancer patients were at severe risk of malnutrition (Stage C, Table 2). Furthermore, the GLIM tool demonstrated a similar rate of severe malnutrition risk (31.55%). However, the GNRI and CONUT nutritional risk scores identified lower rates of severe nutritional risk (6.98% and 0.61%, respectively, Table 1).

The CONUT score presented specificity above 97.2% for predicting various outcomes, including 100% for overall complications, major complications (Clavien-Dindo ≥ 3), longer length of stay (LOS), and higher mortality, as well as 97.2% for advanced disease stage. The specificity of GNRI ranged from 89.7% to 97.1% for these outcomes, with specificities of 97.1% for overall complications, 91.6% for major complications, 98% for longer LOS, 93.9% for higher mortality, and 89.7% for advanced disease stage. The highest specificity of the PG-SGA and GLIM tools was 81.6% and 78.9%, respectively, for predicting advanced disease stage. The CONUT score had a positive predictive value (PPV) of 100% for predicting overall complications, major complications, longer LOS, and higher mortality, while the GNRI had a PPV of 91.7% for predicting longer LOS. Additionally, the PG-SGA showed 55.6% sensitivity for predicting mortality in upper gastrointestinal cancer patients. GLIM exhibited the highest predictive ability for LOS among upper gastrointestinal cancer patients (AUC 0.558) compared to the PG-SGA (AUC 0.528), GNRI (AUC 0.463), and CONUT (AUC 0.495), although its clinical significance remains questioned (Table 4).

## 4. Discussion

The findings of the present study revealed significant variability in the identification of increased risk for severe malnutrition across these tools, as well as in their classification performance metrics (sensitivity, specificity, PPV, NPV, and AUC) across different patient groups and outcomes.

For CRC patients, the CONUT and GNRI tools demonstrated low rates of increased risk for severe malnutrition, whereas the PG-SGA and GLIM tools identified higher rates. Additionally, the CONUT score demonstrated high specificity and PPV for predicting adverse postoperative outcomes, suggesting its effectiveness in correctly identifying non-at-risk patients while minimizing the risk of false positives [36]. In contrast, the GNRI demonstrated high specificity but low sensitivity, possibly missing at-risk for postoperative complications patients, potentially leading to under-treatment. The GLIM and PG-SGA tools exhibited moderate to high specificity, indicating that they are fairly reliable in excluding non-at-risk patients for adverse postoperative outcomes, although not as high as CONUT. This pattern of higher specificity and lower sensitivity was consistent across morbidity and mortality outcomes. The AUC for malnutrition assessment tools varied, with CONUT and GNRI showing moderate discriminative power, suggesting that these tools can accurately distinguish between patients who will and will not develop postoperative complications. This finding aligns with the literature reports of moderate AUC values for these tools in similar patient populations [37].

Similarly, in upper GI cancer patients, the CONUT and GNRI tools demonstrated low rates of increased risk for severe malnutrition, whereas the PG-SGA and GLIM tools identified higher rates of increased risk. The CONUT score maintained high specificity and PPV for complications, major complications, LOS, advanced disease stage, and mortality, indicating its effectiveness in correctly identifying non-at-risk patients while minimizing the risk of false positives [26]. GNRI exhibited a similar pattern with high specificity but low sensitivity. Both GLIM and PG-SGA showed high specificity, indicating their effectiveness in identifying patients at low risk for adverse postoperative outcomes. However, their sensitivity was compromised, which means these tools might miss some patients who are at high risk for adverse postoperative results. The AUC values for these tools reflected moderate to good discriminative ability, comparable to other studies reporting AUCs for nutritional screening tools in upper GI cancer patients [29].

For HPB cancer patients, the CONUT tool demonstrated low rates of increased risk for severe malnutrition during preoperative nutritional assessment, while the PG-SGA and GLIM tools identified higher rates of increased risk. However, the GNRI tool showed the highest rate of increased risk for severe malnutrition in this group. The CONUT score presented high specificity, consistent with its performance in other cancer types, making it very useful for identifying patients who are not at risk of complications. Similarly, the GNRI demonstrated high specificity, indicating a strong ability to rule out non-at-risk patients. GLIM and PG-SGA exhibited moderate to high specificity but demonstrated better sensitivity for mortality prediction compared to other tools, suggesting their utility in identifying at-risk patients, even though they have a higher rate of false positives. The AUC values for CONUT and GNRI in this patient group indicate moderate discriminative power, consistent with previous findings reported for these tools in the literature [38].

Based on the different components of these four tools, the CONUT and GNRI consistently showed low rates of increased malnutrition risk in CRC, upper GI, and HPB cancer patients, while PG-SGA and GLIM identified higher risk rates. CONUT in particular demonstrated high specificity and PPV across all cancer types, making it effective in identifying non-at-risk patients and minimizing false positives. GNRI followed a similar pattern with high specificity but lower sensitivity, meaning it may miss some at-risk patients. In contrast, PG-SGA and GLIM had moderate to high specificity but lower sensitivity, making them reliable in excluding non-at-risk patients, though they may overlook some who are at risk. The AUC values for CONUT and GNRI indicated moderate discriminative power, supporting their usefulness in predicting postoperative complications. Meanwhile, PG-SGA and GLIM showed better sensitivity for predicting mortality, suggesting their utility in identifying patients at higher risk, despite a higher rate of false positives. Overall, CONUT and GNRI excelled in ruling out adverse postoperative results’ risk, while PG-SGA and GLIM were more effective at identifying those at risk, especially for mortality.

The high specificity and positive predictive value (PPV) observed for CONUT and GNRI across all patient groups and outcomes highlight their strength in confirming the absence of malnutrition-related adverse postoperative results. While the lower sensitivity of these tools is a critical consideration, the clinical significance of their high specificity and PPV should not be underestimated. Tools with high specificity minimize false positives, which is crucial for avoiding overestimation of risks and associated costs. Our findings align with previous studies that have reported high specificity for CONUT in predicting postoperative complications in gastrointestinal cancer and acute heart failure patients [37,39]. Similarly, the GNRI has been reported to have high specificity in identifying malnutrition in elderly cancer patients [29,38]. On the other hand, despite their lower specificity compared to CONUT and GNRI, GLIM and PG-SGA can be very helpful in identifying non-at-risk patients, which is consistent with findings from other studies [19,20,21,22,23]. Interestingly, GLIM and PG-SGA identified higher rates of patients at increased risk of malnutrition compared to CONUT and GNRI in this study. This suggests their superiority as malnutrition assessment tools, likely due to their more comprehensive assessment criteria [40,41,42,43].

**Figure 1:**
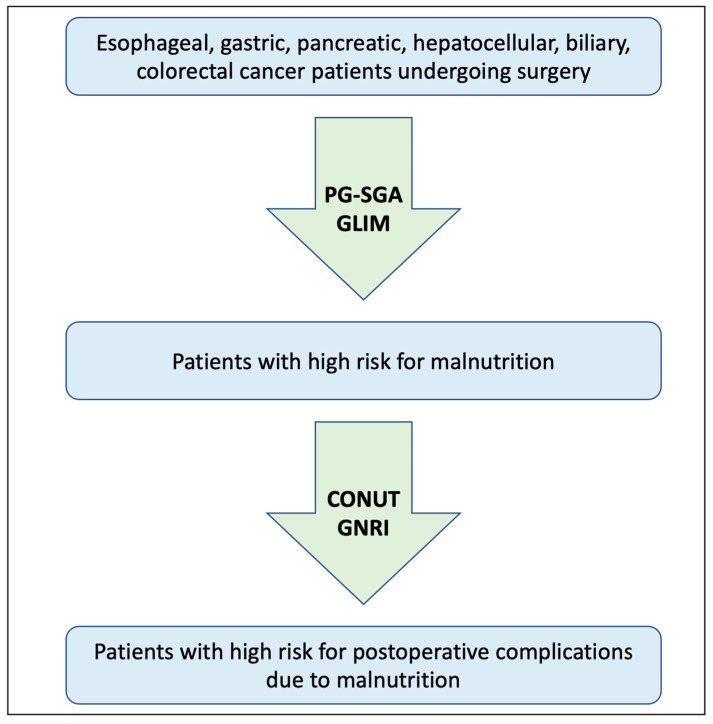
The step-up approach of preoperative nutritional assessment for patients undergoing surgery due to gastrointestinal cancer. CONUT, Controlling Nutritional Status; GLIM, Global Leadership Initiative on Malnutrition; GNRI, Geriatric Nutritional Risk Index; PG-SGA, Patient-Generated Subjective Global Assessment.

Under these circumstances, the findings of the present study confirm the current recommendations of international clinical nutrition societies, such as the European Society for Clinical Nutrition and Metabolism (ESPEN), about the preoperative nutritional assessment of patients undergoing surgery due to gastrointestinal cancer using one or more screening tools [44]. Taking into account the great superiority of PG-SGA and GLIM on identifying severe risk for malnutrition among patients undergoing surgery due to colorectal, HPB, and upper GI cancer, along with the high accuracy of CONUT and GNRI in predicting patients who are not going to demonstrate postoperative complications due to malnutrition, as proved by the present study, we recommend a step-up approach for the preoperative assessment of these patients: The first step of nutritional assessment should be based on PG-SGA or GLIM tools, and after determining patients in high risk, the potential therapeutic interventions which could affect the postoperative outcomes due to malnutrition should be designed for patients presenting high risk for complications according to the evaluation with CONUT or GNRI tools for each cancer type (Figure 1). In that way, nutritional therapeutic interventions for patients who do not actually need them will be avoided, saving resources and providing timely operations without the risk of oncologic burden for patients. However, the accuracy of these nutritional tools needs to be validated in diverse clinical settings and patient populations. Moreover, a continuous assessment of these tools will help in refining cut-offs, making the stratification even more accurate. This necessitates the inclusion of nutritional assessment into the broader perioperative care framework for gastrointestinal cancer patients undergoing surgery, involving dietitians, surgeons and oncologists, finally enhancing patient outcomes.

Future research should focus on developing and validating new or combined screening approaches that maintain high specificity while improving sensitivity. Such efforts will ensure that all at-risk patients are accurately identified and managed, ultimately improving postoperative outcomes and quality of life for cancer patients. Additionally, further investigation should be conducted using different categories within malnutrition screening tools and scoring systems to enhance the sensitivity of the tools, while maintaining balanced prognostic ability. Finally, a combination of validated malnutrition assessment tools, such as PG-SGA with GLIM criteria should be evaluated to determine the cut-off points that enhance their prognostic ability concerning postoperative results.

To the best of our knowledge, this is the first study to investigate the prognostic abilities of two malnutrition assessment tools (PG-SGA and GLIM) and two nutritional scoring systems (CONUT and GNRI) concerning postoperative outcomes. The prospective design of the study allowed for real-time data collection and monitoring, reducing recall bias and enhancing the accuracy of postoperative outcome assessments. Additionally, including patients with various cancer types from two major university hospitals increased the generalizability of the findings. The detailed reporting of patient characteristics and postoperative outcomes provided a comprehensive view of the impact of malnutrition on surgical outcomes. Surgical operations were performed by the same team of surgeons at each center, and nutritional assessments were conducted by the same group of dietitians using identical tools, ensuring consistency and homogeneity in the data. The study’s sample size also contributed to a post hoc power of 85%. Finally, the use of predefined inclusion criteria minimized selection bias and ensured a well-defined patient population.

However, this study had certain limitations. Despite including patients from two hospitals, single-center biases may still affect generalizability. Additionally, monitoring patients for at least 30 days post-discharge may not capture the long-term nutritional impact on postoperative outcomes. Finally, there are limited studies directly comparing the performance of these specific nutritional screening tools in predicting postoperative outcomes, making direct comparisons challenging.

## 5. Conclusions

This study highlights the variability and strengths of current malnutrition screening tools across different cancer patient groups and postoperative outcomes. Tools like CONUT and GNRI exhibit high specificity, making them invaluable for confidently ruling out non-at-risk patients and minimizing false positives. In contrast, tools like GLIM and PG-SGA, while slightly less specific, provide a balanced approach that can complement high-specificity tools to enhance overall screening efficacy. Therefore, a step-up approach is recommended for preoperative nutritional assessment in patients undergoing surgery for gastrointestinal cancer, starting with malnutrition assessment using PG-SGA and GLIM, followed by the complementary evaluation and design of preoperative nutritional interventions with CONUT or GNRI. This approach should be validated in future studies and tested across different clinical settings and diverse patient populations.

## Figures and Tables

**Table 1:** Patient characteristics.

	Colorectal Cancer	Hepatobiliary Cancer	Upper GI Cancer
Number of patients	205	103	172
Age (years ± SD)	69.2 ± 11.7	63.8 ± 12.4	66.6 ± 11.2
Gender (%)			
Male	62	67	67
Female	38	33	33
Body Mass Index (BMI) (%)			
Normal	33.67	35.71	39.29
Overweight	37.69	42.86	38.69
Obese	28.64	21.43	22.02
Eastern Cooperative Oncology Group (ECOG) status (%)			
0	59.9	51	55.8
1	22.1	23.5	24.2
2	8	12.2	9.7
3	9	10.2	7.9
4	1	3,1	2.4
American Society of Anesthesiologists (ASA) score (%)			
1	29.9	24.5	28
2	40.2	37.3	46.4
3	22.1	29.4	15.5
4	7.8	8.8	10.1
Nutritional Assessment			
PG-SGA (%)			
Stage A and B	78.5	75.76	70.24
Stage C	21.5	24.24	29.76
GNRI (%)			
No risk—moderate risk (≥82)	95.1	10.78	93.02
Major risk (<82)	4.9	89.22	6.98
GLIM (%)			
Normal and moderate risk	80.9	77.32	68.45
Severe risk	19.1	22.68	31.55
CONUT (%)			
Normal to moderate (score 0–8)	98.94	96.71	99.39
Severe (score ≥ 9)	1.06	3.29	0.61
Complications (%)			
Yes	34.65	69.6	43.45
No	65.35	30.4	56.55
Clavien-Dindo classification (%)			
<3	63.8	54.3	47.36
≥3	36.2	45.7	52.64
Stage (%)			
<III	57.06	38.2	52.7
≥III	42.94	61.8	47.3
Length of stay (LOS, %)			
<7 days	21.57	10	30.3
≥7 days	78.43	90	69.7
Mortality			
Yes	1.95	9.7	5.23
No	98.05	90.3	94.77

ECOG, Eastern Cooperative Oncology Group Performance Status scale; ASA, American Society of Anesthesiologists Physical Status classification; CONUT, Controlling Nutritional Status; GLIM, Global Leadership Initiative on Malnutrition; GNRI, Geriatric Nutritional Risk Index; PG-SGA, Patient-Generated Subjective Global Assessment.

**Table 2:** Predictive values of malnutrition tools for patients undergoing surgery due to colorectal cancer.

	Sensitivity	PPV	Specificity	NPV	AUC
Complication severity (Clavien-Dindo ≥ 3)					
CONUT	4.2	50	97.5	62.9	0.492
GLIM	16	33.3	81.8	63.2	0.517
GNRI	12	60	95.5	65.6	0.45
PG-SGA	28	46.7	81.8	66.7	0.454
Overall complications					
CONUT	3.1	100	100	66.5	0.484
GLIM	17.1	31.6	79.4	63.3	0.51
GNRI	7.1	50	96.2	66	0.473
PG-SGA	21.4	34,9	78	64.3	0.490
Length of stay (≥7 days)					
CONUT	1.3	100	100	21.8	0.493
GLIM	18.5	76.3	78	20	0.502
GNRI	5.6	90	97.7	21.8	0.483
PG-SGA	23.4	86	85.4	22.4	0.441
Mortality					
CONUT	0	0	98.9	97.9	0.505
GLIM	0	0	80.5	97.5	0.601
GNRI	50	20	96	99	0.258
PG-SGA	50	4.7	79.1	98.7	0.356
Stage (≥III)					
CONUT	3.2	100	100	58,3	0.484
GLIM	26.6	51.5	81.8	60.5	0.465
GNRI	9.1	75	97.8	59.2	0.473
PG-SGA	36.9	66.7	86.4	65	0.378

CONUT, Controlling Nutritional Status; GLIM, Global Leadership Initiative on Malnutrition; GNRI, Geriatric Nutritional Risk Index; PG-SGA, Patient-Generated Subjective Global Assessment; PPV, positive predictive value; NPV, negative predictive value; AUC, area under the curve.

**Table 3:** Predictive values of malnutrition tools for patients undergoing surgery due to HPB cancer.

	Sensitivity	PPV	Specificity	NPV	AUC
Complication severity (Clavien-Dindo ≥ 3)					
CONUT	6.7	66.7	96.8	51.7	0.482
GLIM	22.6	50	80	53.8	0.492
GNRI	9.4	37.5	86.5	52.5	0.498
PG-SGA	34.4	68.8	85.7	58.8	0.391
Overall complications					
CONUT	4.8	100	100	32.2	0.475
GLIM	20.9	63.6	72.4	28.4	0.533
GNRI	12.9	81.8	93.5	32.2	0.478
PG-SGA	23.5	66.7	73.3	29.7	0.517
Length of stay (≥7 days)					
CONUT	1.3	50	90	10.5	0.543
GLIM	20.2	81	60	8.2	0.588
GNRI	11.2	90.9	90	10.2	0.517
PG-SGA	20.9	78.3	50	6.8	0.632
Mortality					
CONUT	20	66.7	98.8	90.9	0.395
GLIM	55.6	22.7	80.7	94.7	0.328
GNRI	20	18.2	90.2	91.2	0.414
PG-SGA	50	20.8	78.7	93.3	0.341
Stage (≥III)					
CONUT	3.3	100	100	34.1	0.482
GLIM	19.4	54.5	70.6	32.4	0.560
GNRI	12.1	50	80	35.6	0.498
PG-SGA	18.2	60	76.5	32.5	0.526

HPB, hepato-pancreatic-biliary; CONUT, Controlling Nutritional Status; GLIM, Global Leadership Initiative on Malnutrition; GNRI, Geriatric Nutritional Risk Index; PG-SGA, Patient-Generated Subjective Global Assessment; PPV, positive predictive value; NPV, negative predictive value; AUC, area under the curve.

**Table 4:** Predictive values of malnutrition tools for patients undergoing surgery due to upper GI cancer.

	Sensitivity	PPV	Specificity	NPV	AUC
Complication severity (Clavien-Dindo ≥ 3)					
CONUT	2.7	100	100	47.8	0.486
GLIM	34.2	61.9	75.8	50	0.449
GNRI	7.9	75	97.1	49.3	0.459
PG-SGA	34.2	61.9	76.5	51	0.434
Overall complications					
CONUT	1.4	100	100	56.3	0.493
GLIM	29.6	39.6	65.6	55	0.534
GNRI	5.5	33.3	91.6	55.8	0.507
PG-SGA	29.2	42	68.5	55.3	0.524
Length of stay (≥7 days)					
CONUT	0.9	100	100	28.4	0.495
GLIM	29.5	64.7	63.3	28.2	0.558
GNRI	9.6	91.7	98	32	0.463
PG-SGA	28.8	66.7	68	30.1	0.528
Mortality					
CONUT	11.1	100	100	95.1	0.444
GLIM	44.4	7.5	69.2	95.7	0.441
GNRI	22.2	16.7	93.9	95.6	0.409
PG-SGA	55.6	10	71.7	96.6	0.369
Stage (≥III)					
CONUT	0	0	97.2	50	0.514
GLIM	21.2	46.7	78.9	53.6	0.505
GNRI	8.6	42.9	89.7	52.2	0.527
PG-SGA	33.3	61.1	81.6	58.5	0.431

GI, gastrointestinal; CONUT, Controlling Nutritional Status; GLIM, Global Leadership Initiative on Malnutrition; GNRI, Geriatric Nutritional Risk Index; PG-SGA, Patient-Generated Subjective Global Assessment; PPV, positive predictive value; NPV, negative predictive value; AUC, area under the curve.

## Data Availability

All data are available upon reasonable request.

## Supplementary Materials

The following supporting information can be downloaded at: https://www.mdpi.com/article/10.3390/jcm13237479/s1. Table S1. Phenotypic and etiologic criteria for the diagnosis of malnutrition according to the GLIM criteria. Table S2. Thresholds for severity grading of malnutrition into Stage 1 (Moderate) and Stage 2 (Severe) malnutrition. Table S3. Assessment of undernutrition degree by CONUT.
