# Supplementary material for: The Predictive Role of Preoperative Malnutrition Assessment in Postoperative Outcomes of Patients Undergoing Surgery Due to Gastrointestinal Cancer: A Cross-Sectional Observational Study
Source: J Clin Med. 2024 Dec 9;13(23):7479. doi: 10.3390/jcm13237479 (PMC11642265; doi:10.3390/jcm13237479)
Supplement: Supplementary file 1 [file jcm-13-07479-s001.zip › jcm-3330180-supplementary.pdf]

**Supplementary Table S1.** Phenotypic and etiologic criteria for the diagnosis of malnutrition according to the GLIM criteria.				
Phenotypic Criteria [g]			Etiologic Criteria [g]	
Weight loss (%)	Low body mass index (kg/m^{2})	Reduced muscle mass [a]	Reduced food intake or assimilation [b,c]	Inflammation [d-f]
>5% within past 6 months,<br>or >10% beyond 6 months	<20 if < 70 years, or<br><22 if >70 years<br>Asia:<br><18.5 if < 70 years, or<br><20 if >70 years	Reduced by validated body composition measuring techniques [a]	≤50% of ER > 1 week, or any reduction for >2 weeks, or any chronic GI condition that adversely impacts food assimilation or absorption [b,c]	Acute disease/injury [d,f] or chronic disease-related [e,f]

GI: gastro-intestinal, ER: energy requirements.

[a] For example fat free mass index (FFMI, kg/m2)) by dual-energy absorptiometry (DXA) or corresponding standards using other body composition methods like bioelectrical impedance analysis (BIA), CT or MRI. When not available or by regional preference, physical examination or standard anthropometric measures like mid-arm muscle or calf circumferences may be used. Thresholds for reduced muscle mass need to be adapted to race (Asia). Functional assessments like hand-grip strength may be considered as a supportive measure.

[b] Consider gastrointestinal symptoms as supportive indicators that can impair food intake or absorption e.g. dysphagia, nausea, vomiting, diarrhea, constipation or abdominal pain. Use clinical judgment to discern severity based upon the degree to which intake or absorption are impaired. Symptom intensity, frequency, and duration should be noted.

[c] Reduced assimilation of food/nutrients is associated with malabsorptive disorders like short bowel syndrome, pancreatic insufficiency and after bariatric surgery. It is also associated with disorders like esophageal strictures, gastroparesis, and intestinal pseudo-obstruction. Malabsorption is a clinical diagnosis manifest as chronic diarrhea or steatorrhea. Malabsorption in those with ostomies is evidenced by elevated volumes of output. Use clinical judgment or additional evaluation to discern severity based upon frequency, duration, and quantitation of fecal fat and/or volume of losses.

[d] Acute disease/injury-related. Severe inflammation is likely to be associated with major infection, burns, trauma or closed head injury. Other acute disease/injury-related conditions are likely to be associated with mild to moderate inflammation.

[e] Chronic disease-related. Severe inflammation is not generally associated with chronic disease conditions. Chronic or recurrent mild to moderate inflammation is likely to be associated with malignant disease, chronic obstructive pulmonary disease, congestive heart failure, chronic renal disease or any disease with chronic or recurrent Inflammation. Note that transient inflammation of a mild degree does not meet the threshold for this etiologic criterion.

[f] C-reactive protein may be used as a supportive laboratory measure.

[g] Requires at least 1 phenotypic criterion and 1 etiologic criterion for diagnosis of malnutrition.

Supplementary Table S2. Thresholds for severity grading of malnutrition into Stage 1 (Moderate) and Stage 2 (Severe) malnutrition.

	Phenotypic Criteria [a]		
	Weight loss (%)	Low body mass index (kg/m2) [b]	Reduced muscle mass [c]
**Stage 1/Moderate Malnutrition** (Requires 1 phenotypic criterion that meets this grade)	5-10% within the past 6 mo, or 10-20% beyond 6 mo	<20 if < 70 yr, <22 if ≥ 70 yr	Mild to moderate deficit (per validated assessment methods - see below)
**Stage 2/Severe Malnutrition** (Requires 1 phenotypic criterion that meets this grade)	>10% within the past 6 mo, or >20% beyond 6 mo	<18.5 if < 70 yr, <20 if ≥70 yr	Severe deficit (per validated assessment methods - see below)

[a] Severity grading is based upon the noted phenotypic criteria while the etiologic criteria are used to provide the context to guide intervention and anticipated outcomes.
[b] Further research is needed to secure consensus reference BMI data for Asian populations in clinical settings.
[c] For example appendicular lean mass index (ALMI, kg/m2) by dual-energy absorptiometry or corresponding standards using other body composition methods like
bioelectrical impedance analysis (BIA), CT or MRI. When not available or by regional preference, physical examination or standard anthropometric measures like mid-arm muscle or calf circumferences may be used. Functional assessments like hand-grip strength may be used as a supportive measure.

**Supplementary Table S3.** Assessment of undernutrition degree by CONUT.				
	Undernutrition Degree			
Parameter	Normal	Light	Moderate	Severe
Serum Albumin (g/dl)	3.5 - 4.5	3.0 - 3.49	2.5 - 2.9	< 2.5
Score	0	2	4	6
Total Lymphocytes/ml	> 1600	1200-1599	800-1199	< 800
Score	0	1	2	3
Cholesterol (mg/dl)	> 180	140-180	100-139	< 100
Score	0	1	2	3
Screening Total Score	0-1	2-4	5-8	9-12
